# The Marshall Complex in the Human Heart: Embryology, Microanatomy, Autonomic Features and Clinical Implications for Atrial Fibrillation—A State-of-the-Art Narrative Review

**DOI:** 10.3390/jcm14238422

**Published:** 2025-11-27

**Authors:** Wojciech Bartosz Dutka, Adam Bochenek, Tomasz Lepich, Marcin Malinowski, Grzegorz Bajor

**Affiliations:** 1 Department of Human Anatomy, Faculty of Medical Sciences in Katowice, Medical University of Silesia, 40-762 Katowice, Poland; adambo8899@gmail.com (A.B.); lepich@sum.edu.pl (T.L.); gbajor@365.sum.edu.pl (G.B.); 2 Department of Cardiac Surgery, Faculty of Medical Sciences in Katowice, Medical University of Silesia, 40-007 Katowice, Poland; mmalinowski@sum.edu.pl; 3 Department of Anatomy, Faculty of Medicine, University of Ostrava, 70300 Ostrava, Czech Republic

**Keywords:** vein of Marshall, oblique vein of the left atrium, ligament of Marshall, left superior vena cava, atrial fibrillation, cardiac anatomy, electrophysiology, ablation, Marshall bundle

## Abstract

The vein and ligament of Marshall (VOM and LOM) are embryological remnants that have gained increasing recognition due to their anatomical complexity, arrhythmogenic potential, and relevance during catheter ablation and structural heart interventions. This review summarizes current evidence on their embryology, morphology, anatomical variability, imaging characteristics, and clinical implications. A structured literature search across PubMed, Embase, and Scopus identified anatomical, histological, electrophysiological, and interventional studies. The VOM is present in most hearts, but its topographic variants and ostial positions show substantial interindividual diversity. The LOM displays a segmental architecture with distinct muscular and fibrotic components that interface with the atrial myocardium and the coronary sinus, providing a substrate for atrial fibrillation. Advances in cardiac imaging have improved delineation of the VOM–LOM region, enhancing pre-procedural assessment and guidance for ethanol infusion and ablation strategies. Recognition of the variability and functional significance of these structures is essential for optimizing procedural outcomes and avoiding complications. Taken together, the VOM and LOM represent key atrial venous remnants whose detailed characterization contributes to a deeper understanding of atrial arrhythmogenesis and contemporary interventional electrophysiology.

## 1. Introduction

The venous system of the human heart originates from a complex embryonic network that undergoes extensive remodeling during fetal development [1]. Within this process, the left superior vena cava (LSVC) normally regresses, leaving behind two complementary remnants: the vein of Marshall (VOM) and the ligament of Marshall (LOM), representing its venous and fibrous components, respectively [2,3]. Failure of this regression may result in a persistent LSVC (PLSVC), the most common thoracic venous anomaly [4,5,6]. A more detailed account of this embryological background is provided in the dedicated section of this review.

**Figure 1:**
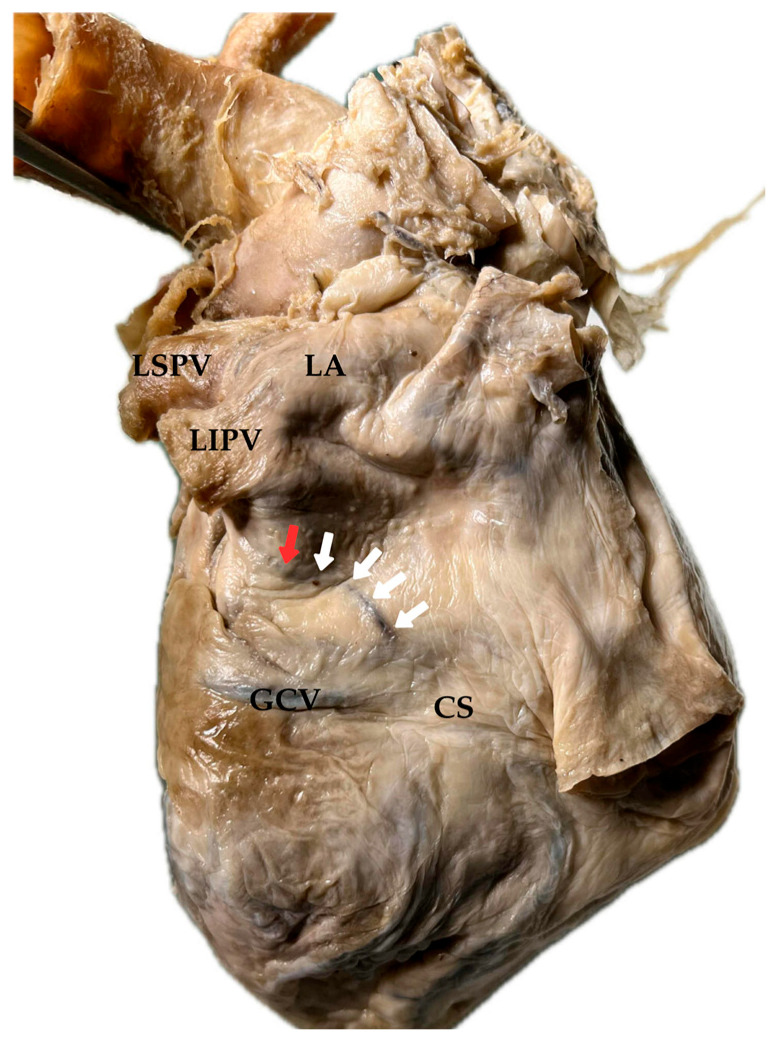
Anatomical view of the left atrial region of the heart. The course of the vein of Marshall (VOM) is indicated by the white arrows, while the red arrow marks its termination. Additional anatomical landmarks are labeled as follows: LA—left atrium, LSPV—left superior pulmonary vein, LIPV—left inferior pulmonary vein, GCV—great cardiac vein, CS—coronary sinus. The image presents the specimen in its original state, with only graphical annotations (arrows and labels) added. The unprocessed full image is available as Supplementary Materials (Figure S1).

First described by John Marshall in 1850, the VOM is a thin-walled vessel draining blood from the posterolateral wall of the left atrium (LA) into the coronary sinus (CS) at its junction with the great cardiac vein (GCV) [3,7,8] (Figure 1). From an anatomical perspective, the LOM is a pericardial fold that may contain the VOM together with fibrous tissue, autonomic fibers, small vessels, and ganglia [3,4,9,10,11]. These developmental insights help explain the marked variability in the presence and morphology of the Marshall structures.

The prevalence of the VOM, as demonstrated in both anatomical and imaging studies, is highly variable and depends on the diagnostic modality used—ranging from 71% to 98% in cadaveric series, approximately 63% with dedicated computed tomography (CT) protocols, and up to 73% in coronary sinus angiography (CSA) [8,12,13,14,15,16,17]. In some cases, the VOM is not identifiable, with only the LOM remaining as a constant anatomical structure that typically follows the same course [8,9,10,11].

Histologically, the LOM consists of myocardial bundles (the Marshall bundles, MB), autonomic fibers—predominantly cholinergic, but also adrenergic—small ganglia, blood vessels, and surrounding fibrofatty tissue [9,10,18,19]. Owing to its muscular and neural components, the LOM is considered a potential source of ectopic electrical activity initiating atrial arrhythmias, including paroxysmal atrial fibrillation (AF) [9,10,11,18,19]. Similar properties have also been attributed to the VOM, as its wall may contain thin extensions of atrial myocardium (myocardial sleeves), small ganglia, and autonomic fibers, enabling both impulse conduction and modulation of atrial electrical activity [10,11,12,19,20].

In recent years, novel anatomical and electrophysiological insights—particularly regarding pulmonary vein incorporation into the LA and the embryological link between the Marshall structures and PLSVC—have underscored their morphological variability and clinical relevance [4,5,6,21].

The clinical importance of the VOM and LOM arises from their role in atrial arrhythmogenesis and their value as therapeutic targets. Numerous studies have demonstrated that both structures participate in the initiation and maintenance of AF, making them critical areas of interest during ablation procedures [10,11,22,23,24,25,26]. Ethanol infusion into the vein of Marshall (EIVOM) has been shown to facilitate mitral isthmus block and improve arrhythmia-free survival, as confirmed in randomized controlled trials (VENUS) and prospective, though single-center, studies (Marshall-PLAN) [24,26].

Understanding the anatomical variability, embryological origin, and clinical implications of the Marshall structures is therefore of particular importance for cardiologists, electrophysiologists, and anatomists. To date, most studies have addressed either their developmental aspects or their clinical applications in isolation. The present review aims to provide a comprehensive synthesis of current knowledge on the morphology, embryology, and function of the VOM and LOM, with special emphasis on their role in AF pathophysiology and in therapeutic strategies targeting arrhythmogenic substrates.

## 2. Materials and Methods

### 2.1. Search Strategy

This work was designed as a narrative, state-of-the-art review. Therefore, the PRISMA framework was not applied, although elements of structured literature selection were included to ensure transparency. Searches were performed in PubMed, Scopus, and ScienceDirect, as well as in open-access repositories (e.g., Google Scholar). No time restrictions were applied, allowing the inclusion of classical works of historical significance, including Marshall’s original description from 1850. The literature search was conducted between August and December 2024. The search strategy was based on Boolean combinations of the following keywords: “vein of Marshall”, “ligament of Marshall”, “oblique vein of the left atrium”, “left superior vena cava”, “atrial fibrillation”, “cardiac embryology”, “cardiac anatomy”, “Marshall bundle”, “ethanol ablation”, “coronary sinus anatomy”, “persistent LSVC”. Eligible sources comprised peer-reviewed articles and selected classical works of recognized scientific importance. No language restrictions were applied, except for excluding studies for which the full text was not accessible.

### 2.2. Inclusion and Exclusion Criteria 

Eligible studies comprised original anatomical, histological, imaging, and clinical investigations related to the VOM and LOM, as well as case reports providing procedural or illustrative data, high-quality review articles, and classical works of embryological or anatomical importance. Excluded were unpublished or non–peer-reviewed papers, articles without full-text availability, and duplicates across databases. Studies not directly addressing the anatomy, embryology, or clinical relevance of the Marshall structures, as well as those of low methodological quality or with imprecise source data, were also excluded. 

### 2.3. Selection of Publications and Data Extraction

The selection process was performed in two stages. In the first stage, two authors independently screened titles and abstracts, excluding studies inconsistent with the scope of the review. In the second stage, the full texts of the remaining publications were assessed, and any disagreements were resolved by consensus. Only studies meeting the predefined eligibility criteria and judged methodologically sound were included. The selected publications were thematically grouped into four main categories: embryological development of the Marshall structures, anatomical variability (morphometric and topographic), clinical significance and arrhythmogenic role, and imaging or interventional techniques related to this region.

### 2.4. Study Selection Summary

A total of 243 publications were identified: 215 from electronic databases and 28 from other sources (e.g., bibliographies of cited works, repositories). After removal of 57 duplicates, 186 unique records were screened. Of these, 112 were excluded based on title and abstract as irrelevant to the scope of the review. Full-text assessment was conducted for 85 articles, of which 17 were excluded due to low quality, lack of source data, or overly general content. Ultimately, 68 publications met the eligibility criteria and were included in this review. 

### 2.5. Ethical Considerations

No new studies involving humans or animals were performed by the authors. Figure 1 presents an original photograph of a human heart specimen obtained from the Department of Anatomy collection, Medical University of Silesia. The specimen was derived from the university’s body donation program and was used in accordance with institutional ethical standards. The photograph is shown in its original form without any digital modification of the tissue. Only graphical annotations (arrows and labels indicating anatomical landmarks) and a uniform white background were added for clarity. The unprocessed full image with the original background has been provided as Supplementary Materials (Figure S1). All donors participating in the institutional body donation program had provided written informed consent.

## 3. Embryological Origin of the Vein and Ligament of Marshall

In the earliest embryonic stages, the venous system is arranged symmetrically and formed by paired anterior and posterior cardinal veins. The anterior cardinal veins drain the cranial region of the embryo and are connected by the transverse precardinal anastomosis, while the posterior cardinal veins return blood from the caudal part. Both sets of veins converge into the common cardinal veins (ducts of Cuvier), which empty into the primitive sinus venosus [1,2]. 

During subsequent development, the left anterior cardinal vein together with the left common cardinal vein give rise to the LSVC. Around the seventh gestational week, the left brachiocephalic vein forms, redirecting blood flow to the right side, which ultimately leads to regression of the LSVC [1,27].

**Figure 2:**
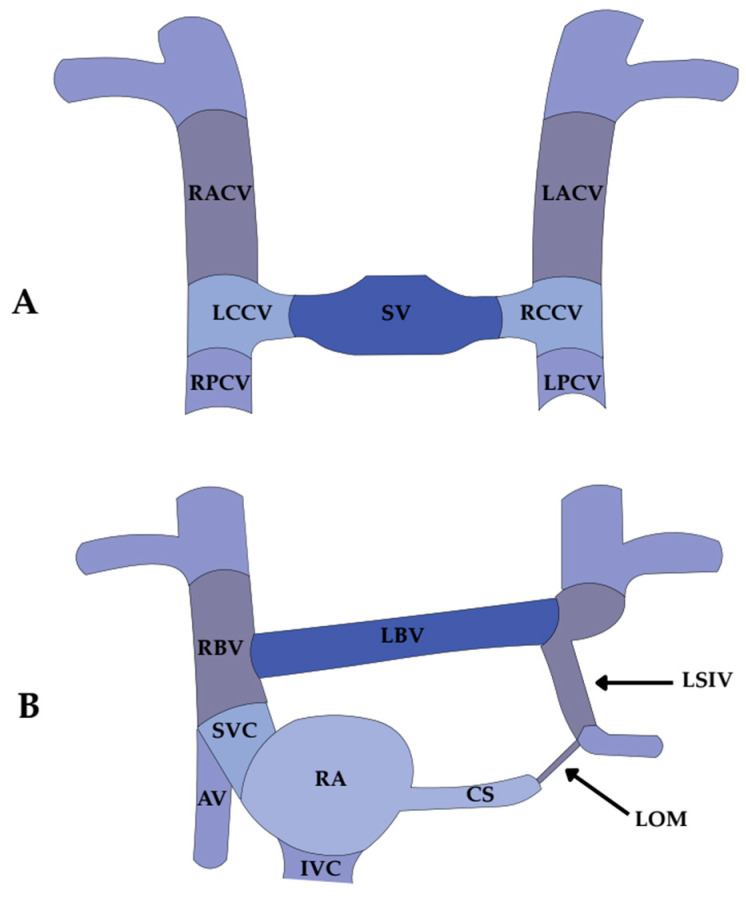
(**A**) Embryonic venous system with paired anterior cardinal veins (RACV, LACV) and posterior cardinal veins (RPCV, LPCV), draining via common cardinal veins (RCCV, LCCV) into the sinus venosus (SV). Regression of the left superior vena cava (LSVC) occurs after formation of the left brachiocephalic vein (LBV). (**B**) Ligament of Marshall (LOM) containing the vein of Marshall (VOM) draining into the coronary sinus (CS). Other labeled structures: right atrium (RA), superior vena cava (SVC), inferior vena cava (IVC), azygos vein (AV), right brachiocephalic vein (RBV), and left superior intercostal vein (LSIV).

After regression, the LSVC leaves two distinct remnants: a fibrous remnant known as the LOM, first described by John Marshall in 1850 [3], and a venous remnant that persists as the VOM, draining into the CS near the valve of Vieussens. The left horn of the sinus venosus with the adjacent part of the left common cardinal vein develops into the CS (Figure 2).

**Figure 3:**
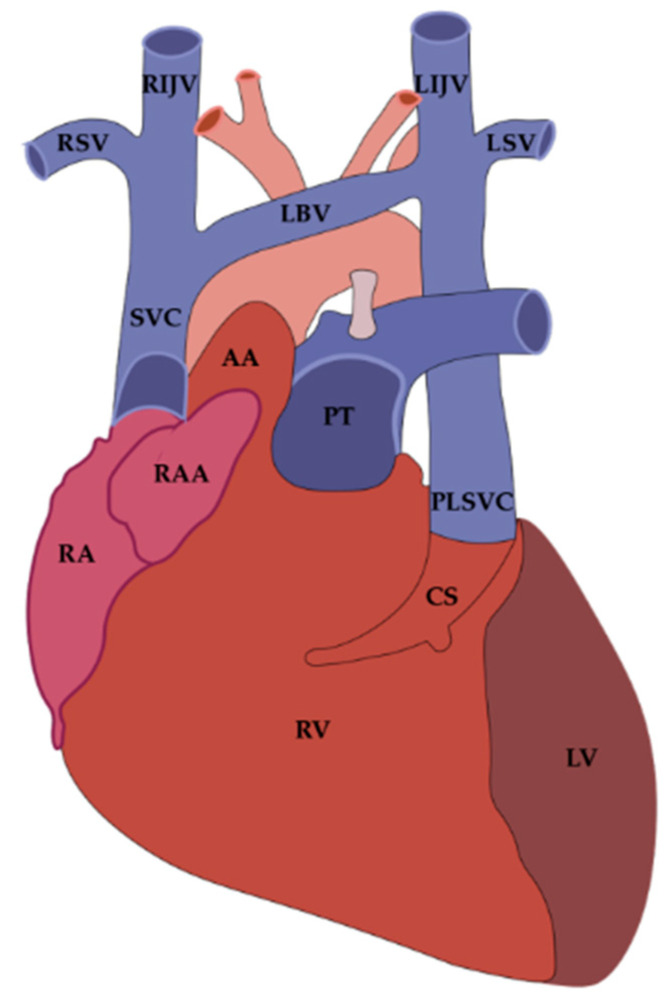
Schematic representation of a persistent left superior vena cava (PLSVC) draining into the coronary sinus (CS). Other relevant venous and cardiac structures are also labeled, including the right atrium (RA), right atrial appendage (RAA), right ventricle (RV), left ventricle (LV), ascending aorta (AA), pulmonary trunk (PT), superior vena cava (SVC), left brachiocephalic vein (LBV), right subclavian vein (RSV), left subclavian vein (LSV), right internal jugular vein (RIJV), and left internal jugular vein (LIJV).

Thus, the VOM and LOM are morphologically distinct but developmentally linked, representing different outcomes of LSVC regression: the VOM as a small patent intracardiac remnant (traditionally attributed to the left anterior or common cardinal vein, depending on the embryological perspective), and the LOM as its fibrous counterpart. Failure of LSVC regression results in a PLSVC, the most frequent thoracic venous malformation, reported in approximately 0.5–3% of the general population and up to 11% of individuals with congenital heart disease. The coexistence of PLSVC with the VOM reflects their shared embryological origin [4,5,6] (Figure 3). The coexistence of VOM and PLSVC is embryologically consistent, and in this setting, the VOM may appear more prominent and easier to visualize angiographically, although direct evidence for this association remains limited [4,5,6]. Moreover, incorporation of pulmonary veins (PVs) into the LA during fetal life may further influence the final morphology of the VOM and LOM [21].

Gray’s Anatomy describes the LOM as a fibrous strand extending from the highest left intercostal vein toward the left atrium, continuing along the course of the VOM [28]. This description highlights the superior attachment of the LOM to the left superior intercostal vein and its topographic relation to the left brachiocephalic vein. However, many anatomical studies describe only the intracardiac component (VOM) and its connection to the CS, without further detailing the extracardiac superior attachment of the LOM.

Importantly, understanding the embryologic continuum between the LSVC, VOM and LOM provides valuable insights into the clinical significance of a PLSVC. Because PLSVC represents the persistence of embryonic structures that normally regress into the Marshall complex [6,29,30], its presence alters the architecture of the CS and surrounding atrial tissues. These anatomical changes are frequently associated with CS dilatation and displacement of adjacent conduction pathways, which may contribute to arrhythmogenesis [31,32,33].

In particular, the persistence of LSVC-derived structures may give rise to aberrant electrical conduction pathways and facilitate the aggregation of autonomic ganglia within the Marshall complex, thereby linking this developmental anomaly to both structural and neurogenic substrates of AF [11,18,19,34].

Moreover, when the PLSVC contains myocardial sleeves, it may act as a substrate for ectopic activity, in line with clinical evidence indicating that the PLSVC can serve as a trigger or driver of AF in a substantial proportion of patients [35]. Thus, the same embryologic processes that give rise to the VOM and LOM also establish the structural and electrophysiological framework through which the PLSVC may influence atrial conduction and promote arrhythmogenesis.

## 4. Anatomical Course and Morphological Variability of the Vein and Ligament of Marshall

The morphological variability of the VOM and the LOM arises from differences in their embryological development, which is reflected both in their anatomical structure and their clinical significance. The VOM usually originates at the junction of the GCV and CS, running obliquely through the epicardial fat between the left atrial appendage (LAA) and the left pulmonary veins (LPVs) [3,7,8,13]. The LOM is consistently observed as a fibrous remnant of the regressed LSVC. If the VOM is not patent, the course of the LOM still indicates its expected trajectory [9,10,11]. Anatomically, the LOM extends from the highest intercostal vein and left brachiocephalic vein across the posterolateral wall of the LA, before joining the terminal portion of the VOM at its entry into the CS [9,10,28]. This variability reflects the degree of LSVC regression during embryological development. 

### 4.1. Presence and Prevalence of the Vein and Ligament of Marshall

Anatomical variability of the VOM and LOM has important implications for the course and effectiveness of AF ablation procedures, including EIVOM [7,8,9,13,19,36,37]. In part of the population, the VOM may be completely absent or obliterated, and its prevalence strongly depends on the diagnostic method applied.

In cadaveric studies, including micro-CT analyses, the prevalence of VOM identification has been reported as high, ranging from 71% to 98%. The highest values were reported by DeSimone et al. [12] (98%) and Cendrowska-Pinkosz & Urbanowicz [8] (97%), whereas the lowest prevalence was observed by Żabówka et al. [13] [(71%). Intermediate findings were provided by de Oliveira et al. [37] (87%) and Delgove et al. [17] (84%). These data indicate that, in anatomical investigations, the VOM is present in the majority of cases, although its presence and course demonstrate substantial interindividual variability.

In clinical imaging, the detection rate of the VOM is considerably lower, ranging from approximately 20% to 73%. In conventional CT, the prevalence was 20.3% in the series by Młynarski et al. [16] and 35% in the study by Takagi et al. [15]. Higher detection rates were reported with dedicated VOM-CT protocols (63%) [15] and with CSA, where Ding et al. [14] observed a prevalence of 73%. These discrepancies highlight the importance of diagnostic modality when planning ablation procedures. An additional challenge is the presence of a PLSVC, which may obscure or complicate differentiation of the VOM from adjacent venous structures [4,5,6,38].

The prevalence of the LOM has not yet been reliably established in large, unselected cadaveric series. Available evidence suggests, however, that it is a constant or nearly constant structure. Makino et al. [19] reported the presence of MB in 89% of hearts (25/28), whereas Kim et al. [9] identified the LOM in all seven specimens analyzed. Nevertheless, comprehensive population-based anatomical studies lack definitive prevalence estimates (Table 1).

### 4.2. Morphological Variations in the Vein and Ligament of Marshall

The substantial anatomical variability of the VOM has led to two main approaches to its classification described in the literature: (A) based on the branching pattern and tributaries, and (B) based on the anatomical length and extent. Such categorization is clinically relevant, as it may help to anticipate potential difficulties in identifying and cannulating the VOM and to plan interventional strategies more precisely [8,17].

#### 4.2.1. Classification Based on Branching Pattern/Tributaries (Cendrowska-Pinkosz & Urbanowicz)

**Figure 4:**
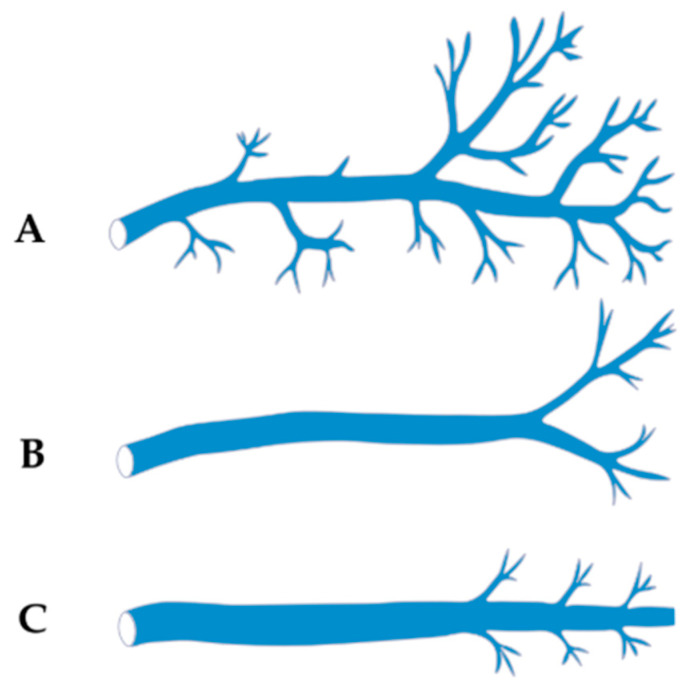
Schematic representation of the three branching types of the vein of Marshall (VOM) according to the classification proposed by Cendrowska-Pinkosz and Urbanowicz. (**A**) Dendritic type—numerous fine tributaries converging into a single trunk draining into the coronary sinus; (**B**) Forked type—two main tributaries merging into a single vein without additional lateral branches; (**C**) Simple type—a straight vessel lacking both initial tributaries and distal side branches.

In the classical cadaveric corrosion-cast study of 100 hearts performed by Cendrowska-Pinkosz and Urbanowicz [8], the VOM could not be identified in three cases (3%). Therefore, the classification of branching patterns was based on 97 specimens. Three principal branching types of the VOM were distinguished (Figure 4):

- Dendritic Type—characterized by numerous fine tributaries converging into a single trunk draining into the CS. The length of the common trunk ranged from 0.5 to 1.8 mm, and this type was found in approximately 17.5% of cases.
- Forked Type—defined by the presence of two main tributaries merging into a single vein at a distance of 0.5–1.3 mm below the point of connection, without additional lateral branches. This was the most common variant, observed in about 48.5% of cases.
- Simple Type—lacking both initial tributaries and distal side branches, identified in approximately 34% of cases.
- In some cases, the VOM forms a plexiform structure or consists of multiple tiny channels, complicating its visualization in imaging and hindering successful cannulation during procedures [14,17].

#### 4.2.2. Classification Based on Length and Anatomical Extent (Delgove et al.)

**Figure 5:**
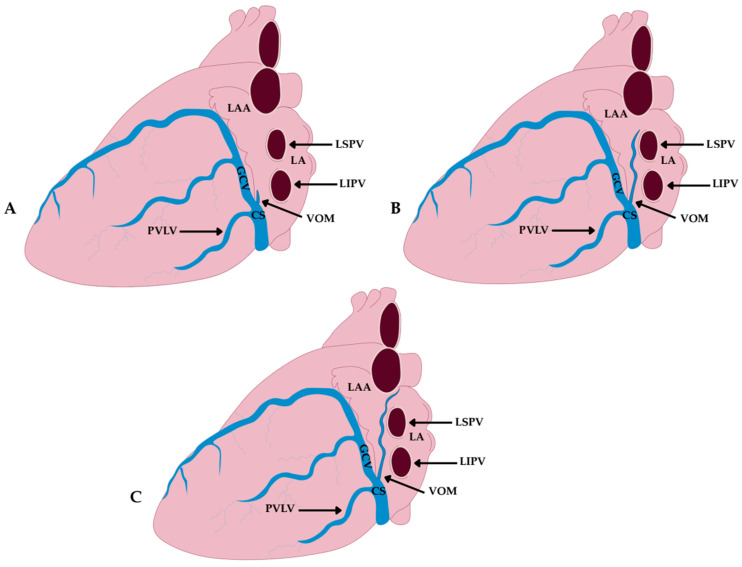
Schematic representation of the three length/anatomical-extent types of the vein of Marshall (VOM) according to Delgove et al.: (**A**) Short Type—terminates within the coronary sinus (CS), without further extension toward the left atrium (LA); (**B**) Intermediate Type—extends up to the posterior wall of the LA; (**C**) Long Type—runs all the way to the roof of the LA. Other relevant venous and cardiac structures are also labeled, including the left atrial appendage (LAA), left superior pulmonary vein (LSPV), left inferior pulmonary vein (LIPV), posterior vein of the left ventricle (PVLV), coronary sinus (CS), and left atrium (LA).

In a micro-CT analysis of 18 hearts, a three-tier classification of the VOM course was proposed [17] (Figure 5):

- Short Type—terminating within the CS, without further extension toward the LA; observed in 16% of cases.
- Intermediate Type—extending up to the posterior wall of the LA; found in 57% of cases.
- Long Type—running all the way to the roof of the LA; present in 27% of cases.

Additionally, in 20–33% of specimens, accessory tributaries originating from the posterior atrial wall or perivenous region were noted. These may be of clinical importance, providing potential arrhythmogenic foci and influencing the efficacy of ablation techniques [17].

In response to this anatomical complexity, some groups have introduced the concept of the oblique vein and ligament area (OVLA), encompassing not only the main trunk of the VOM but also its lateral tributaries and adjacent tissue. This extended definition may better reflect the functional–anatomical unit relevant to arrhythmogenesis, potentially improving diagnostic accuracy in imaging and guiding ablation procedures [17].

Overall, the morphological diversity of the VOM—and, in its absence, the topographical overlap of the LOM along its typical course—has significant practical implications. Multibranching venous patterns may reduce the effectiveness of interventions and complicate imaging-based identification, underscoring the need for individualized strategies in ablation procedures [8,9,14,15,16,17,19]. Unlike the VOM, no morphological classification of the LOM has yet been established. Although it is considered a nearly constant structure, current studies have primarily focused on its histological composition and arrhythmogenic potential rather than proposing distinct anatomical subtypes [9,10,19].

### 4.3. Size and Dimensions of the Vein and Ligament of Marshall

The VOM exhibits considerable morphometric variability. In classical anatomical descriptions, it is reported as a structure measuring approximately 1 mm in diameter and 20–30 mm in length, but cadaveric and imaging studies demonstrate a much wider range of values. In the study by Żabówka et al. [13] he mean length of the VOM was 30.8 ± 13.6 mm (range 9–72 mm), with significant differences depending on the pattern of pulmonary venous drainage: in hearts with the classical pattern, the length averaged 27.6 ± 10.4 mm, whereas in variant patterns it was markedly greater (48.3 ± 5.6 mm). In the micro-CT analysis by Delgove et al. [17] VOM length ranged from 12.3 to 72.2 mm (mean 36.5 ± 19.4 mm). 

In cadaveric studies, de Oliveira et al. [37] reported a mean VOM ostial diameter of 1.23 ± 0.38 mm and a distance from the CS ostium of 30.9 ± 10.2 mm. DeSimone et al. [12] described a mean distance from the CS ostium of 24 ± 4 mm and a mean patent segment length of 9.3 ± 6.6 mm. In another anatomical study by Ortale et al. [36] the VOM had a diameter ranging from 0.4 to 1.8 mm (mean 1.0 ± 0.4 mm). 

In clinical imaging, shorter VOM segments were generally observed compared with cadaveric studies. In conventional cardiac CT, Młynarski et al. [16] reported a mean visible segment length of 9.24 ± 7.58 mm and a diameter of 1.72 ± 0.69 mm. In the study by Takagi et al. [15] the distance from the VOM ostium to the CS ostium ranged from 22 to 52 mm (mean 36 ± 7 mm), while the vessel diameter ranged from 1.1 to 2.6 mm (mean 1.6 ± 0.3 mm). In CSA, Ding et al. [14] demonstrated that patients with AF had a larger VOM ostial diameter (1.9 ± 0.9 mm) compared with individuals without AF (1.7 ± 0.7 mm), suggesting a possible link between this feature and the pathogenesis of AF. Hypothetically, a larger VOM caliber may facilitate ectopic conduction or increase susceptibility to reentry, making it an attractive therapeutic target for ablation and chemical denervation. 

In cases where the VOM is absent as a patent venous structure, its typical course may be represented by the LOM. However, due to the absence of a vascular lumen and the presence of surrounding fibrofatty tissue, the LOM is more difficult to assess morphometrically. To date, no reliable reference values for its length or “diameter” have been established—available studies provide only qualitative descriptions [9,10]. High interindividual variability, along with differences in preparation and imaging techniques, further complicates the standardization of measurements (Table 2).

### 4.4. Topography and Ostium of the Vein and Ligament of Marshall

**Figure 6:**
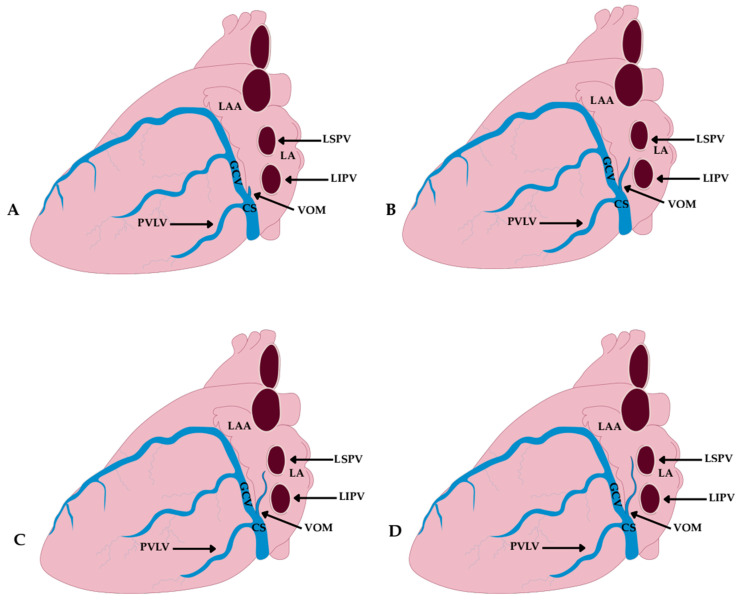
Schematic representation of the four topographic types of the vein of Marshall (VOM) based on its vertical extent relative to the left pulmonary veins: (**A**) Type I—terminating below the left inferior pulmonary vein (LIPV); (**B**) Type II—reaching the level of the LIPV; (**C**) Type III—ascending to the intervein area; (**D**) Type IV—extending up to the left superior pulmonary vein (LSPV). Other relevant venous and cardiac structures are also labeled, including the left atrial appendage (LAA), left atrium (LA), coronary sinus (CS), and posterior vein of the left ventricle (PVLV).

A cadaveric study by Żabówka et al. [13] on 200 human hearts identified four topographic types of the VOM, classified according to its vertical extent in relation to the LPV (Figure 6):

- Type I—terminating below the left inferior pulmonary vein; observed in 21.9% of cases.
- Type II—reaching the level of the left inferior pulmonary vein; observed in 47.7% of cases.
- Type III—ascending to the intervein area; observed in 17.2% of cases.
- Type IV—extending up to the left superior pulmonary vein; observed in 13.3% of cases.

This topographic classification has direct procedural implications, particularly in planning vascular access and target localization during ablation procedures. A high course of the VOM (types III–IV) may facilitate access to intervein structures and the posterior wall of the LA but at the same time increases the risk of damaging adjacent structures [13]. 

In their corrosion-cast study, Cendrowska-Pinkosz and Urbanowicz [8] also proposed a classification of the VOM ostium within the CS, distinguishing four types:

- Group A—ostium at the level of the posterior vein of the left ventricle and the GCV opening; observed in 24% of cases.
- Group B—ostium at the level of the posterior vein of the left ventricle; 11% of cases.
- Group C—ostium at the level of the GCV; 7% of cases.
- Group D—independent ostium, separate from other tributaries of the CS; the most common variant, seen in 58% of cases.

This classification emphasized that the VOM ostium most frequently opens independently, which has practical implications for CS cannulation and electroanatomical mapping. 

The ostium of the VOM is typically located near the proximal portion of the CS, often in close proximity to the Vieussens valve [17,37,39]. Micro-CT analysis by Delgove et al. [17] confirmed that the venous ostium is almost always situated proximally to this valve, making it a reliable anatomical landmark during cannulation. In their series, the Vieussens valve was present in 46.9% of cases with a VOM, and in 90% of instances the venous ostium was located closer to the CS ostium than to the valve itself. While the valve leaflet may sometimes obscure the ostium, creating technical difficulties during cannulation, it can also serve as a stabilizing support for guidewires or catheters [39]. Shah et al. [4] also reported variability in the ostial position and emphasized technical challenges in VOM cannulation, suggesting that adjacent anatomical structures may be used to aid localization in imaging [4].

The LOM exhibits a segmental arrangement (proximal, mid, and distal portions) that differ in their muscular connections with the CS, the LPV, and the atrial wall [10]. Hwang and Chen [10] highlighted that its predictable anatomical course is of particular value when planning ablation strategies targeting the posterior atrial wall. 

Taken together, both the VOM and LOM—despite their distinct structural nature—represent embryological remnants with relatively predictable trajectories. This consistency allows them to serve as reliable anatomical landmarks during interventional procedures, including EIVOM and CS cannulation. 

## 5. Histological and Functional Structure of the Vein and Ligament of Marshall 

The VOM and LOM represent complex structures composed of muscular, neural, vascular, and fibro-fatty components. They are therefore not merely embryological remnants but actively participate in atrial conduction and modulation. Their histological composition favors both the initiation and maintenance of atrial arrhythmias through the creation of preferential conduction pathways, integration of autonomic inputs, and maintenance of metabolically active arrhythmogenic substrates. Understanding the detailed histological architecture and its functional implications is essential for clarifying their role in the pathogenesis of AF and other atrial tachyarrhythmias (AT), as well as for designing effective therapeutic strategies [9,10,11,20,23]. 

### 5.1. Muscular Components of the Vein and Ligament of Marshall

Both the LOM and the VOM may contain myocardial elements that play a central role in atrial conduction and arrhythmogenesis [9,11,12,19,22]. 

The LOM is characterized by the presence of MB—strands of striated atrial myocardium occurring singly or in multiple fascicles. These bundles run superficially to the VOM or within connective tissue, often intersecting at different levels [9,11,19]. Preparatory studies and micro-CT imaging have demonstrated substantial variability in the number, configuration, and spatial relationships of MB to the VOM [12,17,19]. In human cadaveric hearts, the average length of MB was ~7.8 ± 3.9 mm, with a mean diameter of 0.7 ± 0.2 mm [9]. 

Classic anatomical work by Hwang and Chen [10] subdivided the LOM into three segments:

- Proximal segment—connected to the muscular sleeve of the CS, serving as a potential conduction pathway between the CS and the MB.
- Middle segment—extending toward the LA ridge and forming connections with the left PV.
- Distal segment—running superiorly above the PV and in some cases reaching the free wall of the LA.

Makino et al. [19] demonstrated that in one-third of studied hearts, multiple and extensive connections existed between the LOM and the LA, highlighting the marked morphological variability of this structure. This segmental organization provides the substrate for complex reentrant circuits. 

MB establish muscular connections with the CS musculature, the free LA wall, the LAA, and the PV sleeves. Such multifocal connectivity increases conduction heterogeneity and predisposes to atrial arrhythmogenesis [9,11,12,19]. Histological staining (H&E, Masson’s trichrome) confirmed that MB are separated from surrounding structures by collagenous septa and embedded within fibro-fatty tissue [9,19]. In animal models, Yu et al. [40] demonstrated connexin-43 (Cx43) expression within the LOM, potentially relevant to intercellular coupling and conduction stability. However, reliable evidence for connexin-40 (Cx40), atrial natriuretic peptide (ANP), or caveolin-3 in MB is lacking; these findings have been largely limited to the sinoatrial node, representing a major gap in current knowledge [41].

Electrophysiologically, MB conduct impulses bidirectionally between the CS and the LA. In vivo mapping has delineated three major connection patterns: CS–LOM, LA–PV via the LOM, and multifocal CS–LA–PV networks [10,11,42,43]. The highest arrhythmogenic potential is observed in configurations with multiple connections, where fractionated potentials and reentry phenomena are frequently recorded [9,10,11,19].

Under physiological conditions, MB contribute to synchronization of LA activation during sinus rhythm and influence P-wave morphology on the surface electrocardiogram (ECG) [10,11]. In pathological states, however, they may serve as a substrate for reentry and focal automaticity, particularly under enhanced adrenergic drive [9,18]. In animal models, “double potentials” have been recorded—one arising from the atrial myocardium and the other from the LOM—confirming its ability to conduct independently [18].

In cases where the VOM remains patent, its wall may contain thin myocardial sleeves continuous with the LA musculature [12,19]. These sleeves provide direct conduction between the CS and LA and may participate in reentrant circuits. The autonomic innervation of the VOM will be addressed in the following subsection. 

### 5.2. Nerve Components of the Vein and Ligament of Marshall

The VOM and LOM are densely integrated with the cardiac autonomic nervous system and play a central role in conduction and modulation of both parasympathetic and sympathetic inputs [9,10,11,18,19,22]. Immunohistochemical studies have demonstrated the presence of cholinergic fibers (ChAT/AChE-positive) and adrenergic fibers (TH-positive) [18,19]. The mean cholinergic-to-adrenergic fiber ratio is approximately 12.6:1 (±3.9), confirming the predominance of parasympathetic innervation [18].

The distribution of autonomic fibers follows a distinct topographic pattern. In the proximal LOM (near the CS ostium), cholinergic fibers predominate, modulating atrial effective refractory period (ERP) and conduction stability. In contrast, the distal portion, extending toward the PV, contains a higher density of adrenergic fibers [10,18,19]. Cholinergic fibers are abundant within the adipose tissue surrounding the MB and in proximity to the LAA, CS musculature, and posterior atrial fat pad [9,18,19]. Adrenergic fibers, though less numerous, form dense clusters within adipose tissue and course predominantly along vascular structures, particularly in distal segments [18,19].

Functionally, sympathetic activation enhances automaticity and promotes focal ectopy within the LOM/MB, whereas parasympathetic activation shortens the ERP across atrial regions, facilitating the initiation and maintenance of AF [10,11,18,22]. Concomitant stimulation of both parasympathetic and sympathetic branches further increases dispersion of refractoriness in the LA, exacerbating electrical instability and favoring reentry circuits [18,19].

The LOM also acts as a parasympathetic conduit for branches of the left vagus nerve. Ablation of the LOM attenuates vagally induced ERP shortening in remote regions of the LA [18]. Clinically, LOM ablation reduces atrial response to vagal stimulation, underscoring its pivotal role in parasympathetic modulation and its contribution to the pathogenesis of AF [11,19,22,40].

### 5.3. Nerve Ganglia of the Vein and Ligament of Marshall

Neural ganglia located within the VOM and LOM represent clusters of neurons capable of receiving, processing, and integrating autonomic inputs, acting as local centers of cardiac modulation [10,11,19]. These ganglia influence both atrial automaticity and the conduction properties of the myocardium [10,11,19].

The largest cholinergic ganglia (ChAT+/AChE+) are concentrated near the junction of the VOM with the CS, with their density gradually decreasing toward the PV [18,19]. In contrast, adrenergic structures (TH+) show the opposite distribution, with greater density in the distal portion of the LOM, closer to the PV ostia [18,19]. These ganglia are frequently situated adjacent to MB, vagal fibers, and myocardial sleeves of the VOM, allowing them to directly modulate conduction within atrial structures [9,18,19,22].

In some specimens, ganglia have been identified within the adventitial layer of the VOM, suggesting their ability to modulate conduction not only within the vein itself but also in its atrial connections [10,18,19]. The coexistence of both cholinergic and adrenergic components within these ganglia confirms their role in dual modulation of atrial activity—parasympathetic and sympathetic—acting synergistically or antagonistically depending on physiological conditions [10,11,18,19]. 

Functionally, LOM/VOM ganglia integrate signals from preganglionic and postganglionic fibers, contributing to the regulation of sinoatrial node activity and other atrial structures [10,18]. High-frequency stimulation (HFS) applied to the LOM/VOM region and adjacent ganglionated plexi can provoke atrial arrhythmias as well as vagal reflexes [10,11,40]. 

From a clinical perspective, ablation targeting these ganglia reduces atrial responsiveness to vagal stimulation and decreases the inducibility of AF, highlighting their critical role in arrhythmogenesis [18,25,40]. 

### 5.4. Vascular Components of the Vein and Ligament of Marshall

Within the LOM, a dense network of accompanying vessels has been described, including arterial branches supplying the MB, autonomic fibers, and the surrounding fibro-fatty tissue, as well as small venous tributaries draining into the VOM or directly into the CS [9,37]. This vascular arrangement provides the morphological basis for local modulation of excitability and conduction in the LOM/VOM region.

The lumen of the VOM is of particular clinical importance: it can be used for EIVOM, enabling the elimination of conduction along connections within the LOM/mitral isthmus, as well as for diagnostic maneuvers and mapping during electrophysiological procedures [24,25,37]. However, due to the rich vascularization of this area, there is a risk of local bleeding or iatrogenic injury during catheter manipulation and ablation—a risk that can be minimized through appropriate preprocedural imaging and meticulous procedural technique [9,15,25].

### 5.5. Fibro-Fatty Tissue of the Ligament of Marshall

Fibro-fatty tissue represents the dominant structural substrate of both the LOM and VOM, encompassing MB, autonomic fibers, and vessels, while clearly separating these elements from the adjacent atrial myocardium [9,12]. Histological studies have shown that MB are embedded within a fibro-fatty matrix, which often insulates them from the LA muscle, creating tissue barriers and corridors that favor directional conduction [9,10,12,18,19]. This organization facilitates the formation of specialized conduction pathways with arrhythmogenic potential in the LOM/VOM region [9,10,18,19]. 

Although the specific collagen composition of the LOM matrix has not yet been comprehensively analyzed, it is well established that type I and type III collagens dominate within atrial tissue, influencing wall stiffness, electrical remodeling, and conduction properties [44,45]. It is likely that these same isoforms prevail in the LOM, reinforcing both anatomic barriers and conduction channels in this area. 

The adipose component surrounding MB contains autonomic fibers and microvessels, serving not only a protective function for conducting structures but also a metabolic role for local neurons and muscle fibers [9,18,19]. Dense capillary networks and numerous cholinergic and adrenergic fibers have been documented penetrating the fibro-fatty matrix, further augmenting the arrhythmogenic significance of this region [9,18,19]. 

From an interventional standpoint, the variable thickness and composition of fibro-fatty tissue in the LOM/VOM can limit effective energy transfer during ablation. Both radiofrequency and cryoenergy demonstrate reduced penetration in the presence of thick fat layers, as confirmed in studies of epicardial adipose tissue surrounding the PV, where abundant fat attenuated ablation efficacy and promoted AF recurrence [46,47]. Clinically, this necessitates tailoring procedural strategies, adjusting energy parameters, or considering alternative techniques [15,30]. Optimized imaging protocols, including dedicated CT planning for EIVOM, may further reduce the risk of procedural failure and complications [15].

## 6. The Importance of the Vein and Ligament of Marshall in Atrial Fibrillation

Atrial fibrillation is the most prevalent sustained arrhythmia globally and its burden continues to rise, affecting millions of individuals, particularly older adults and patients with underlying cardiovascular disease [48,49]. It is characterized by rapid, irregular, and chaotic atrial activation, leading to the loss of effective atrial contraction and impaired ventricular filling. This carries major clinical implications, as AF increases the risk of stroke, heart failure, and other adverse cardiovascular outcomes [50,51]. 

Both the LOM and the VOM are integral to the complex pathophysiology of AF. As previously detailed, these structures contain a combination of myocardial bundles and autonomic fibers [9,18,19]. Clinically, this dual myogenic–neurogenic composition creates a substrate capable of generating ectopic impulses and facilitating reentry [10,20,22,25]. Myocardial strands within the LOM can conduct impulses directly to the LA wall and CS, while autonomic inputs—particularly cholinergic activation—shorten atrial refractoriness and promote arrhythmia initiation [10,18,19]. When the VOM is patent, myocardial sleeves along its wall provide additional conduction pathways between the CS region and the LA, further lowering the threshold for AF initiation [12,37]. Collectively, these properties explain why the Marshall complex serves as both a trigger of AF and an attractive but technically challenging ablation target [20,22,24,25,43]. 

Electrophysiological mapping studies have demonstrated that the LOM/VOM region can generate rapid, fractionated activity and focal discharges, functioning as potent triggers for AF episodes [10,11,22,42,43]. Their close anatomical connections with the posterior LA wall, the mitral isthmus, and the PV facilitate the formation of reentry circuits and the maintenance of AF [12,22,37,43]. 

The coexistence of muscular and neural components in this region enables bidirectional interactions: autonomic activation may provoke rapid discharges within the musculature, while local myogenic activity can modulate autonomic input, creating a self-perpetuating arrhythmogenic mechanism [10,18,19,22]. 

In summary, the VOM and LOM constitute specialized myo-autonomic complexes that can act both as independent arrhythmogenic foci and as integral components of larger reentrant circuits. Their electrophysiological properties, combined with their strategic anatomical location, make them critical structures in the initiation and maintenance of AF and other AT [9,12,18,19,22,24,25,37].

## 7. The Role of the Vein and Ligament of Marshall in Other Atrial Arrhythmias

Beyond their well-documented role in the pathophysiology of AF, the VOM and LOM are increasingly recognized as important contributors to the mechanisms of other atrial arrhythmias [14,16,26,52,53].

### 7.1. Perimitral Atrial Flutter (PMFL)

In PMFL, the VOM and LOM may serve as epicardial conduction bridges linking reentrant circuits to the LA [43,53]. Clinical observations and mapping studies have demonstrated that the VOM and its associated epicardial musculature can enable conduction around the mitral annulus, allowing the reentry circuit to bypass an endocardial mitral isthmus block [11,43,53]. Consequently, targeting the VOM during ablation can facilitate the achievement of durable mitral isthmus block [25]. 

### 7.2. Focal Atrial Tachycardia (FAT)

Both the LOM and VOM have been identified as potential sources of FAT independent of the PV [10,11]. The presence of myocardial fibers, including myocardial sleeves within the VOM, together with dense autonomic innervation, provides a substrate for triggered activity and micro-reentry [9,11,18,19]. The structural basis for this arrhythmogenic potential, particularly the MB, has also been highlighted in histological studies [9]. 

### 7.3. Post-AF Ablation Atrial Tachycardia

In cases of recurrent atrial tachycardia following AF ablation, the region of the MB is often implicated as a residual arrhythmogenic source or hidden conduction pathway, especially when epicardial connections persist and circumvent endocardial ablation lines [11,23,43]. In such scenarios, VOM-targeted strategies—including EIVOM or ablation—may help eliminate residual conduction and stabilize mitral isthmus block [24,26].

## 8. Ablation Strategies Targeting the Vein and Ligament of Marshall in the Treatment of Atrial Arrhythmias

Catheter ablation, primarily performed as pulmonary vein isolation (PVI), is one of the most effective strategies for the treatment of AF, particularly in patients refractory to pharmacological therapy [49,54]. Within this broader context, ablation targeting the VOM and LOM may provide additional benefit by eliminating ectopic foci, interrupting epicardial conduction pathways, and modulating local autonomic innervation [10,19,23,26,55].

### 8.1. EIVOM 

EIVOM is especially useful in persistent AF, particularly in cases refractory to conventional approaches [24,25,26,56]. The procedure involves selective cannulation of the VOM followed by infusion of 98% ethanol, resulting in chemical necrosis of the MB, autonomic fibers, and accompanying microvasculature. Its mechanisms of action include the following:

1. Permanent interruption of conduction within the MB and epicardial connections [24,25,43].
2. Autonomic denervation by eliminating cholinergic and adrenergic fibers and local ganglia, thereby reducing atrial susceptibility to autonomic triggers [11,18,19,22,25].
3. Occlusion of the VOM lumen and its branches, stabilizing the ablative effect [15,25,57].

Clinically, EIVOM provides access to deep epicardial connections resistant to conventional radiofrequency (RF) ablation, particularly across the mitral isthmus and CS [22,24,25,43]. Prospective trials, including the VENUS randomized controlled trial, demonstrated increased arrhythmia-free survival in persistent AF and facilitated achievement of durable mitral isthmus block, even in patients with prior failed RF ablations [22,24,25,26]. Hybrid strategies combining EIVOM with PVI have shown enhanced efficacy [24,26]. Anatomical and micro-CT studies confirm that ethanol ablation affects not only the main trunk of the VOM but also its branches and adjacent autonomic ganglia within the LOM [15,17,22,25,37,57].

### 8.2. Endocardial RF Ablation

RF ablation represents the standard energy source for endocardial lesion formation and remains the primary strategy for the treatment of atrial arrhythmias [49,54]. Its success depends on creating complete and durable conduction block; incomplete lesion lines predispose to recurrence, particularly in the form of PMFL [11,44,53]. In clinical practice, ablation is most often performed across the mitral isthmus or the atrial “roof line,” with the goal of interrupting reentry circuits and modifying arrhythmogenic substrates.

Mapping from the CS or directly from the VOM can improve precision by facilitating detection of Marshall-related potentials and by confirming conduction block continuity [14,23,37,42,58]. Contemporary electroanatomic mapping systems (CARTO/EnSite) further enhance lesion accuracy and validation, which is particularly important in the mitral isthmus region and near the VOM ostium [59,60].

Achieving a durable mitral isthmus block solely by the endocardial RF approach remains challenging due to epicardial MB running along the GCV and VOM, which can bypass endocardial lesion lines and sustain macro–reentrant circuits [43,55]. This explains the occurrence of recurrent PMFL in the presence of VOM/LOM connections—scenarios in which endocardial RF ablation alone may be insufficient [11,43,53]. Recent studies have shown that pulsed field ablation (PFA) may overcome several limitations of thermal energy in the mitral isthmus region. A multicenter investigation reported that PFA can achieve durable lesions across the mitral isthmus and coronary sinus with high efficacy and safety [61]. Given that residual conduction frequently persists through epicardial connections involving the VOM, combining PFA with EIVOM may provide complementary endocardial and epicardial substrate modification, potentially improving the likelihood of durable mitral isthmus block. Supplementing the strategy with EIVOM increases the likelihood of achieving a complete and durable block (through chemical elimination of epicardial connections and autonomic denervation), as confirmed in prospective clinical studies and anatomical investigations (VENUS RCT, Marshall-PLAN) [22,24,25,26].

### 8.3. Epicardial (Pericardial) Ablation

In cases where endocardial access is insufficient, an epicardial approach may be employed to directly target connections along the LOM/VOM and the GCV. While effective in selected patients, the risk profile (e.g., cardiac tamponade) restricts its use to specialized centers [42,43,54,55].

### 8.4. Laser Ablation

Laser-based approaches to the VOM/LOM are still in early experimental stages. Current evidence is limited to preliminary reports, and standard practice remains based on EIVOM and RF [62].

### 8.5. Cryoablation

Cryothermal energy induces tissue necrosis through freezing and is occasionally used to target Marshall-related substrates, particularly where risk of perforation is high or adjacent to sensitive structures. Advantages include controlled lesion depth and lower perforation risk compared with RF. Limitations are reduced efficacy in the presence of thick fibro-fatty tissue and longer application times [46,47,49]. 

### 8.6. Hybrid Techniques and Imaging Support

Combining EIVOM with PVI and supplementary RF lesions yields superior results in persistent AF, as demonstrated in both randomized (VENUS) and prospective single-center (Marshall-PLAN) studies [22,24,25,26]. Procedural planning and guidance are enhanced by multimodal imaging, including CSA, high-resolution CT optimized for EIVOM, and intracardiac (ICE) or transesophageal echocardiography (TEE) [4,14,15,37,38,49,54,58,63] (Table 3).

## 9. Possible Complications of Ablation Procedures 

Although ablation is one of the most effective therapeutic strategies for AF, it is associated with a risk of peri- and post-procedural complications. The most common adverse events include vascular access complications, cardiac perforation and pericardial tamponade, bleeding, stroke or thromboembolic events, injury to adjacent structures, and phrenic nerve palsy. For this reason, careful risk assessment and close post-procedural monitoring are mandatory [49,54]. 

New atrial arrhythmias may occur after ablation, particularly PMFL in cases of incomplete ablation lines or persistent epicardial conduction through the VOM/LOM and the GCV. These present typical causes of recurrence and frequently require re-intervention to achieve durable conduction block [11,43,53]. Inadequate lesion continuity and failure to address epicardial connections predispose to arrhythmia recurrence and the need for repeat procedures [24,26,53]. 

Importantly, standard practice does not associate AF ablation with an increased risk of ventricular arrhythmias; the main concerns remain the aforementioned complications and recurrent AT. Management should strictly follow the recommendations of the European Society of Cardiology (ESC) [49,54].

## 10. Important Clinical Implications of Ablation Procedures 

From a clinical standpoint, ablation targeting Marshall structures represents a valuable adjunct in the management of persistent AF. The addition of EIVOM to standard ablation improves rhythm control and facilitates durable mitral isthmus block, as demonstrated in the VENUS randomized controlled trial and prospective observational studies. An anatomical approach aimed at eliminating MB further enhances these benefits [24,25,26,52]. Effective rhythm control translates into symptom reduction and improved quality of life—the primary goals of AF ablation according to current guidelines [49].

The efficacy of the procedure depends on technical precision (line continuity, confirmed bidirectional block) and proper patient selection. Optimal outcomes require awareness of the role of the VOM and LOM in arrhythmogenesis and the use of advanced imaging support (CSA, CT protocols optimized for EIVOM), which facilitates identification and elimination of conduction pathways sustaining arrhythmias [4,11,14,15,23]. Difficulties in achieving a durable block are often related to epicardial conduction bridges along the GCV/LOM; recognition and interruption of these connections are crucial to preventing recurrence [43,55]. 

## 11. Imaging of the Vein and Ligament of Marshall 

Accurate identification and imaging of the VOM and LOM are essential in planning and performing ablation procedures, particularly in patients with AF [4,11,14,15]. Both structures pose diagnostic challenges due to their small size, anatomic variability, and close proximity to other atrial elements [9,16,17,37]. The VOM, as a vascular structure, can be directly visualized using contrast studies or CT, whereas the LOM, lacking a vascular lumen, can only be recognized indirectly [4,9,14,15,16,37,63,64]. In clinical practice, both non-invasive modalities (CT, TEE) and invasive techniques (CSA, ICE) are employed [4,14,15,24,38,58], alongside advanced functional approaches such as electroanatomical mapping (EAM, e.g., CARTO, EnSite) and intraoperative electrophysiological localization [42,59,60]. Importantly, high-resolution ex vivo studies such as micro-CT provide highly detailed anatomic data but have no direct clinical application, serving instead as a reference point for imaging modalities used in patients [17].

Recent advances in cardiac imaging have substantially improved the ability to visualize the VOM and surrounding atrial venous structures. High-resolution cardiac magnetic resonance (CMR) has been shown to accurately depict the left atrial wall and venous territories involved in EIVOM, demonstrating its value in characterizing the structural substrate and post-procedural remodeling [65]. Modern cardiac computed tomography angiography (CTA) also provides superior spatial resolution and contrast opacification, enabling reliable identification of the VOM and related anatomical variants [16]. Broader studies of the coronary venous system confirm that small venous channels, including the VOM, can be visualized using advanced CT and MR protocols when acquisition parameters are optimized [66]. Furthermore, dedicated VOM-oriented CT acquisition protocols developed for pre-procedural planning of ethanol infusion have significantly increased detection rates and improved assessment of VOM accessibility [15]. These emerging modalities expand the diagnostic capabilities of non-invasive imaging and enhance pre-interventional evaluation of the VOM–LOM region.

### 11.1. CT 

Multidetector cardiac CT has become one of the most effective non-invasive methods for visualizing atrial structures, including the VOM [16,63,64]. As a vascular structure, the VOM may be depicted after contrast administration, but its detection rate is limited. In conventional protocols, visualization rates are only 20.33% (Młynarski) and 35% (Takagi), depending on cardiac phase, vessel diameter, contrast agent, and reconstruction protocol [15,16]. Dedicated VOM-CT protocols improve sensitivity, achieving detection rates of up to 63% (Takagi) [15]. Nevertheless, due to marked morphological variability and the small vessel caliber, VOM visualization in routine CT remains challenging, and often impossible in daily clinical practice [16,63,64]. 

The LOM, lacking a vascular lumen, cannot be directly depicted on CT. However, in selected cases, it may appear as a thin band of soft tissue along its expected course between the CS and the posterior LA wall. This finding, described in the literature as a “subtle thin band of soft tissue on CT,” may suggest the presence of the LOM [9,38,63,64]. Yet, this approach has not been validated, and its diagnostic reliability remains uncertain. 

The limitations of clinical CT become evident when compared with high-resolution ex vivo studies such as micro-CT, which allow highly detailed anatomic assessment of VOM/LOM morphology and variants [17]. Micro-CT consistently demonstrates significant interindividual variability and serves as a valuable reference for interpreting clinical imaging. By contrast, in vivo CT suffers from lower spatial resolution, motion artifacts, and dependence on acquisition protocols. Consequently, the ability to identify these structures on CT largely depends on image quality and operator expertise [15,63,64].

### 11.2. CSA

CSA, performed during electrophysiological procedures, remains the standard intra-procedural and reference invasive technique for visualizing and cannulating the VOM, especially prior to ethanol infusion [14,24,25,58]. By directly opacifying the venous lumen, it enables precise localization of the VOM ostium into the CS, assessment of accessibility, and procedural planning [14,25,58]. In the angiographic study by Ding et al. [14] VOM detection reached 73% in AF patients, but was significantly lower in individuals without arrhythmia, highlighting the influence of clinical context and the superiority of CSA over non-invasive modalities. Zhang et al. [58] further emphasized the importance of morphological assessment and dedicated angiographic protocols for successful identification and cannulation. 

The LOM cannot be visualized with CSA due to the absence of a vascular lumen. While its presence is sometimes inferred (e.g., in cases where the VOM is absent), such identification is indirect, unvalidated, and based on low-level evidence [9,37]. Moreover, even high-quality venography may fail to reveal the VOM if the ostium is obscured by the Vieussens valve, leading to false-negative results and complicating procedural planning [13,39]. Żabówka et al. [39] demonstrated marked variability in valve morphology, underscoring its impact on CS catheterization and VOM access.

### 11.3. ICE and TEE 

Both modalities can indirectly suggest the presence of the VOM by visualizing the dynamic passage of contrast medium after injection into the vein. The appearance of contrast in the LA confirms a functional venous connection between the VOM and the atrium [15,24]. These techniques are also useful for confirming catheter positioning, monitoring ethanol infusion, and detecting complications such as tamponade or esophageal injury [15,24,38]. 

The LOM, lacking a vascular lumen, cannot be directly visualized. Its presence may only be inferred based on characteristic location and tissue echogenicity [9,37]. However, such observations have not been validated and should be considered hypothetical until further evidence is available. Thus, ICE and TEE should be regarded as auxiliary rather than standalone diagnostic tools in the evaluation of Marshall structures.

### 11.4. EAM 

EAM systems such as CARTO and EnSite NavX are widely used during ablation procedures involving the VOM and LOM. By integrating anatomical and bioelectrical data, they enable precise identification of the VOM course, localization of arrhythmogenic sources, verification of conduction block, and improved safety through reduced fluoroscopy exposure. 

Romero et al. [59] demonstrated that EAM significantly reduces fluoroscopy time while maintaining comparable clinical efficacy compared with conventional mapping, particularly during operator training. Similarly, Bazoukis et al. [60] confirmed that 3D mapping improves both efficacy and safety, especially in complex atrial ablations. 

EAM can also be valuable for functionally localizing the LOM, which is often invisible on CT or CSA. Functional identification is based on local electrogram analysis, with VOM/LOM potentials showing earlier activation relative to sinus rhythm and higher amplitude, enabling reliable localization [43]. Moreover, EAM supports planning and guiding EIVOM by accurately localizing the ostium, controlling catheter positioning, and monitoring electrophysiological changes post-infusion [15,57]. 

Newer generations of EAM—incorporating high-density mapping, ICE integration, and fluoroless workflows—promise to enhance the detection and functional identification of Marshall structures. Multimodal integration of imaging and bioelectrical data may provide more precise, safe, and effective ablation strategies in the future. 

### 11.5. Intraoperative Electrophysiological Localization

Unlike advanced 3D mapping (CARTO, EnSite) used in electrophysiology labs, intraoperative electrophysiological localization described by Langmuur et al. [42] is based on point-by-point atrial tissue stimulation during cardiac surgery. By recording local electrograms and tissue responses to pacing, this technique allows identification of the LOM even when it is not visible on CT or CSA. Characteristic signals (high amplitude, early activation relative to sinus rhythm) indicate the functional presence of the LOM and enable tailored ablation strategies. While technically demanding and dependent on operator expertise, this approach provides a valuable adjunct in refractory AF cases where conventional imaging fails (Table 4). 

## 12. Age and Sex-Related Differences in the Vein and Ligament of Marshall

There is a lack of high-quality studies directly and systematically analyzing the relationship between age and the morphology of the VOM/LOM. Available works describe in detail the muscular–autonomic composition and topographic variability of these structures, but do not provide cross-sectional analyses of the type “age vs. morphology” [9,10,11,12,13,17,19,22]. With regard to sex-related differences, anatomic data do not indicate significant differences in the diameter or course of the VOM between women and men. However, clinical studies report variable outcomes and efficacy of strategies involving EIVOM [22,24,25,64].

### 12.1. Age-Related Aspects

Classical anatomical and histological investigations confirm the presence of muscular bundles, autonomic fibers, ganglia, and rich microvasculature within the LOM/VOM. To date, however, no systematic age-dependent changes in these structures have been demonstrated in humans [9,10,11,12,17]. Recent reviews also emphasize a clear evidence gap in this field [10,11,22,23,42]. Hypothetically, aging processes, atrial remodeling, and progressive fibrosis may modulate the electrophysiological role of the Marshall structures, but the lack of morphometric data prevents firm conclusions. 

### 12.2. Sex-Related Aspects

Available anatomical series have not demonstrated sex-specific differences in the diameter or vertical extent of the VOM [12,13,17,37]. In contrast, clinical analyses of patients undergoing EIVOM showed that female sex was associated with lower procedural efficacy and higher risk of arrhythmia recurrence, an observation that persisted even after adjusting for covariates [67]. The mechanism underlying these differences remains unclear and is likely multifactorial, involving atrial remodeling, comorbidities, hormonal factors, and variations in the atrial electrical substrate. Importantly, no consistent morphological sex differences in the VOM/LOM have been confirmed [9,10,12,13,17,23,37]. Potential risk factors may include atrial fibrosis and increased epicardial adipose tissue volume [44,45,46,47]. 

### 12.3. Clinical Implications

When planning ablation, it should be recognized that the absence of demonstrated anatomic sex differences does not exclude significant clinical differences in treatment response. In women, more detailed imaging and substrate mapping, as well as closer long-term follow-up after EIVOM, may be justified based on available data [67]. Currently, no clinical recommendations specifically address age or sex in this context.

## 13. Limitation of Current Knowledge

Despite growing interest in the VOM and LOM, current knowledge remains incomplete. Significant limitations include marked interindividual variability in length, diameter, branching pattern, and tissue composition, which makes it difficult to define a “uniform Marshall anatomy” [8,9,12,15,16,17,37]. The available evidence is also highly heterogeneous, derived from cadaveric dissections, corrosion casts, micro-CT, and clinical CT, which limits comparability and standardization across studies [8,9,12,15,16,17,37]. Clinical imaging shows low sensitivity, with CT detecting the VOM in only 20–35% of patients, while LOM visualization remains indirect and unvalidated; moreover, results are strongly operator- and protocol-dependent [9,15,16,37,63,64]. Clinical evidence on ethanol or RF ablation is restricted to specialized centers and selected populations, reducing the generalizability of findings [22,24,25,26,56]. Finally, systematic analyses addressing the influence of age, sex, or atrial remodeling are lacking, and observational data suggest less favorable EIVOM outcomes in women, though the structural correlates of this discrepancy remain unclear [67]. These limitations highlight the need for standardized methodologies, broader patient representation, and integration of anatomical, histological, and clinical data to better define the role of Marshall structures in atrial arrhythmogenesis.

## 14. Future Directions

Future research on the VOM and LOM should address several key gaps. First, standardized imaging methodologies are needed to improve diagnostic accuracy. Optimized CT protocols and the integration of multimodal approaches with EAM and echocardiography may enhance visualization and procedural planning [15,16,22,58,59,60]. High-resolution morphometric studies, ideally conducted on large-scale cohorts, are required to clarify the impact of anatomical variability on AF pathophysiology [17].

Second, randomized clinical trials should further evaluate the efficacy and safety of EIVOM, particularly in underrepresented groups such as women and elderly patients [22,24,25,26]. The discrepancy between anatomical studies (showing no consistent sex differences) and clinical observations (suggesting less favorable outcomes in women) underscores the need for integrative research combining morphological, histological, and clinical data [63].

Third, the dense cholinergic and adrenergic innervation of Marshall structures highlights their potential as targets for neuromodulatory or pharmacological interventions [9,11,18,19,22,40]. Exploring these approaches may provide alternative or complementary strategies to catheter ablation.

Fourth, the potential role of myocardial damage biomarkers such as Troponin T and NT-proBNP in reflecting structural or electrophysiological activity of the Marshall complex remains largely unexplored. While these biomarkers are well-established indicators of myocardial stress, their utility in identifying arrhythmogenic substrates associated with VOM and LOM requires further investigation. Early evidence suggests that these structures may contribute to atrial stretch or microinjury through their autonomic and myogenic properties [68]. Incorporating biomarker assessment into anatomical and electrophysiological studies could improve risk stratification and enhance the understanding of Marshall-related atrial remodeling.

Finally, emerging energy sources such as cryoablation, laser ablation, and PFA warrant systematic evaluation. These technologies may offer safer and more effective elimination of epicardial connections within the Marshall complex, ultimately improving long-term arrhythmia control [26,46,47,49,53,62].

Considering the growing global burden of AF [48,49], advancing the understanding of the Marshall structures and refining therapeutic approaches targeting these structures represent important future priorities in arrhythmia management.

## 15. Conclusions

The aim of this review was to comprehensively summarize current knowledge on the anatomy, histology, imaging, and interventional significance of the VOM and LOM. Once regarded merely as embryological remnants, these structures are now recognized as complex atrial elements with substantial arrhythmogenic potential. Their unique composition—myocardial bundles, dense autonomic innervation, and fibro-fatty tissue—provides anatomical and functional substrates that contribute to atrial ectopy, macro-reentry, and persistent AF.

Although anatomical and imaging studies have broadened our understanding, marked interindividual variability and the limited sensitivity of non-invasive modalities continue to pose diagnostic and procedural challenges. From a clinical perspective, Marshall-targeted ablation strategies, particularly EIVOM, have demonstrated added value in persistent AF by eliminating epicardial conduction pathways, achieving durable mitral isthmus block, and improving arrhythmia-free survival. Hybrid approaches combining EIVOM with PVI and RF ablation further enhance efficacy, while emerging energy sources may provide safer alternatives in the future.

A precise understanding of Marshall anatomy is essential for procedural planning and outcome optimization. Multimodal imaging, integration of anatomical and electrophysiological data, and individualized ablation strategies are key to advancing treatment. Continued collaboration between anatomists, electrophysiologists, and imaging specialists will be critical to fill existing knowledge gaps and to establish the VOM and LOM as integral components of comprehensive AF management.

## Figures and Tables

**Table 1:** Summary of cadaveric (anatomical) and clinical imaging studies assessing the prevalence of the vein of Marshall (VOM).

Study Type	Author (Year)	Method	Cases (N)	Prevalence of VOM
Cadaveric	Cendrowska-Pinkosz & Urbanowicz (2000) [8]	Gross Dissection	100	97%
Cadaveric	DeSimone et al. (2012) [12]	Gross Dissection	589	98%
Cadaveric	de Oliveira et al. (2007) [37]	Gross Dissection	23	87%
Cadaveric	Żabówka et al. (2020) [13]	Gross Dissection	200	71%
Cadaveric	Delgove et al. (2025) [17]	Micro-CT (ex vivo)	18	18%
Clinical Imaging	Młynarski et al. (2018) [16]	CT (conventional)	300	20,33%
Clinical Imaging	Takagi et al. (2022) [15]	CT (conventional)	132	35%
Clinical Imaging	Takagi et al. (2022) [15]	CT (VOM-CT protocol)	126	63%
Clinical Imaging	Ding et al. (2022) [14]	CSA	290	73%

Abbreviations: CT = computed tomography; CSA = coronary sinus angiography; Micro-CT = micro-computed tomography; VOM-CT protocol = optimized CT protocol for vein of Marshall visualization.

**Table 2:** Summary of cadaveric (including micro-CT) and imaging studies evaluating the length, diameter, and distance from the CS ostium of the vein of Marshall (VOM).

Study Type	Author (Year)	Method	Cases (N)	VOM Length (mm)	VOM Diameter (mm)	Distance from CS Ostium (mm)	Remarks
Cadaveric	Żabówka et al. (2020) [13]	Gross Dissection	142	30.8 ± 13.6 (9–72); classical PV pattern: 27.6 ± 10.4; variant pattern: 48.3 ± 5.6	–	–	*p* < 0.001 for length differences
Cadaveric	Ortale et al. (2001) [36]	Gross Dissection	16	–	1.0 ± 0.4 (0.4–1.8)	–	–
Cadaveric	de Oliveira et al. (2007) [37]	Gross Dissection	20	–	1.23 ± 0.38 (ostial)	30.9 ± 10.2	–
Cadaveric	DeSimone et al. (2012) [12]	Gross Dissection	579	–	–	24 ± 4	Patent segment length: 9.3 ± 6.6
Cadaveric	Delgove et al. (2025) [17]	Micro-CT (ex vivo)	15	36.5 ± 19.4 (12.3–72.2)	–	–	Based on micro-CT segmentation and arborization types
Clinical Imaging	Młynarski et al. (2018) [16]	CT (conventional)	61	9.24 ± 7.58 (visible segment)	1.72 ± 0.69 (segmental)	–	–
Clinical Imaging	Takagi et al. (2022) [15]	CT (VOM-CT protocol)	79	–	1.6 ± 0.3 (1.1–2.6; segmental)	36 ± 7 (22–52)	Dedicated VOM-CT protocol
Clinical Imaging	Ding et al. (2022) [14]	CSA	257	–	AF: 1.9 ± 0.9 (ostial); non–AF: 1.7 ± 0.7	–	*p* < 0.05 for diameter differences

Abbreviations: CS = coronary sinus; CT = computed tomography; CSA = coronary sinus angiography; Micro-CT = micro-computed tomography; PV = pulmonary veins; AF = atrial fibrillation.

**Table 3:** Ablation strategies targeting the vein of Marshall (VOM) and ligament of Marshall (LOM)—summary of evidence and applications.

Strategy	Target/Area	Key Mechanism	When to Consider (Clinical Role)	Efficacy Assessment
EIVOM [22,24,25,26,56,57]	VOM/LOM, epicardial connections, mitral isthmus region	Chemoablation: conduction block, autonomic denervation, branch occlusion	Persistent AF, difficult/unstable mitral isthmus block, failed RF ablation, anatomical strategy	Successful VOM cannulation, “staining” of the vein, bidirectional isthmus block, absence of Marshall potentials
Endocardial RF Ablation [11,14,23,37,42,49,53,54,55,58,59,60]	Mitral isthmus region, atrial roof line, CS/VOM-related potentials	Endocardial RF lesion sets: conduction block, substrate modification; mapping from CS/VOM improves precision and assessment of block continuity	First-line ablation strategy for AF; PMFL prevention/treatment; patients with VOM/LOM-related epicardial connections (often supplemented by EIVOM when block is incomplete)	Achievement of durable bidirectional mitral isthmus block, validated by electroanatomic mapping and CS/VOM potentials; clinical efficacy limited by epicardial conduction bridges
Pulsed Field Ablation [61]	Mitral isthmus, CS region	Non-thermal irreversible electroporation	Persistent AF; mitral isthmus ablation when thermal energy fails; can complement EIVOM for epicardial gaps	Durable mitral isthmus lesions demonstrated; promising synergy with EIVOM
Epicardial ablation [42,43,54,55]	Epicardial connections along LOM/VOM and GCV	Targeted epicardial RF	When endocardial ablation is insufficient; necessary to close epicardial bypass circuits	Durable bidirectional isthmus block; caution due to tamponade risk
Cryoablation [46,47,49]	Vulnerable areas/sites with high perforation risk	Cryothermal necrosis (freezing)	Alternative to RF depending on anatomy and operator experience	Continuous lines without conduction; longer applications required
Laser ablation [62]	Localized damage	Photothermal tissue injury	Preliminary reports only; not a standard for VOM/LOM	–
Hybrid approach (EIVOM + PVI + RF) [22,24,25,26]	Elimination of muscular and autonomic components + completion of lines	Combination of chemoablation (EIVOM) with RF/PVI lines	Persistent/refractory AF, recurrences	Freedom from AF/AT, durable isthmus block, no conduction through VOM/LOM

Abbreviations: AF = atrial fibrillation; CS = coronary sinus; EIVOM = ethanol infusion into the vein of Marshall; GCV = great cardiac vein; PVI = pulmonary vein isolation; RF = radiofrequency; PMFL = perimitral atrial flutter.

**Table 4:** Imaging and mapping modalities of the vein of Marshall (VOM) and ligament of Marshall (LOM)—applications and level.

Method	What It Shows	Clinical Role	Level of Evidence
CT (conventional protocol) [9,15,16,37,63,64]	Partial visualization of the VOM, occasional indirect depiction of the LOM	Anatomic studies, reference for other methods	Ex vivo, experimental
CT (VOM-CT protocol) [15,63,64]	More accurate visualization of the VOM	Procedural planning, limited sensitivity	Clinical, limited
CMR (high-resolution) [65,66]	Visualization of LA wall, venous territories, structural remodeling; occasional depiction of VOM course when optimized sequences used	Structural assessment before/after EIVOM; substrate characterization in AF	Clinical, emerging evidence
CTA (optimized /high-resolution) for venous system [16,65]	Better spatial resolution and contrast opacification; reliable identification of small venous structures including VOM; improved detection of anatomic variants	Pre-procedural VOM assessment; enhanced anatomical mapping	Clinical, supportive
micro-CT [17]	Precise morphology of VOM/LOM, branching patterns, course	Improved ablation planning, assessment of VOM accessibility	Clinical, moderate-quality evidence
CSA [14,24,25,37,58]	VOM ostium, morphology, cannulation	Intra-procedural standard prior to EIVOM	Clinical, high
ICE and TEE [9,15,24,37,38]	Indirect visualization of the VOM (contrast), catheter positioning	Intra-procedural monitoring and complication control	Clinical, supportive
EAM (CARTO, EnSite) [43,59,60]	Functional identification of VOM/LOM (electrogram signals)	Functional identification of VOM/LOM (electrogram signals)	Clinical, adjunctive
Intraoperative Electrophysiological Localization [42]	Functional identification of the LOM based on local electrograms	Surgical adjunct, refractory AF cases	Experimental/clinical case reports

Abbreviations: CT = computed tomography; CMR = cardiac magnetic resolution; CSA = coronary sinus angiography; ICE = intracardiac echocardiography; TEE = transesophageal echocardiography; EAM = electroanatomical mapping; EIVOM = ethanol infusion into the vein of Marshall; AF = atrial fibrillation.

## Data Availability

No new data were created or analyzed in this study. Data sharing is not applicable to this article.

## Supplementary Materials

The following supporting information can be downloaded at https://www.mdpi.com/article/10.3390/jcm14238422/s1; Figure S1: Original, unprocessed photograph of the human heart specimen corresponding to Figure 1 in the main text.

## Abbreviations

The following abbreviations are used in this manuscript:

VOM	Vein of Marshall
LOM	Ligament of Marshall
LSVC	Left Superior Vena Cava
PLSVC	Persistent Left Superior Vena Cava
LA	Left Atrium
GCV	Great Cardiac Vein
CS	Coronary Sinus
CT	Computed Tomography
CSA	Coronary Sinus Angiography
MB	Marshall Bundles
AF	Atrial Fibrillation
EIVOM	Ethanol Infusion into the Vein of Marshall
LSPV	Left Superior Pulmonary Vein
LIPV	Left Inferior Pulmonary Vein
PV	Pulmonary Vein
RACV	Right Anterior Cardinal Vein
LACV	Left Anterior Cardinal Vein
RPCV	Reft Posterior Cardinal Vein
LPCV	Left Posterior Cardinal Vein
SV	Sinus Venosus
LBV	Left Brachiocephalic Vein
SVC	Superior Vena Cava
RA	Right Atrium
IVC	Inferior Vena Cava
AV	Azygos Vein
RBV	Right Brachiocephalic Vein
RAA	Right Atrial Appendage
RV	Right Ventricle
LV	Left Ventricle
AA	Ascending Aorta
PT	Pulmonary Trunk
RSV	Right Subclavian Vein
LSV	Left Subclavian Vein
RIJV	Right Internal Jugular Vein
LIJV	Left Internal Jugular Vein
LAA	Left Atrial Appendage
LPV	Left Pulmonary Vein
OVLA	Oblique Vein and Ligament Area
AT	Atrial Tachyarrhythmias
Cx-43	Connexin-43
Cx-40	Connexin-40
ANP	Atrial Natriuretic Peptide
ECG	Electrocardiogram
ERP	Effective Refractory Period
PMFL	Perimitral Atrial Flutter
FAT	Focal Atrial Tachycardia
PVI	Pulmonary Vein Isolation
RF	Radiofrequency
PFA	Pulsed Field Ablation
ICE	Intracardiac Echocardiography
TEE	Transesophageal Echocardiography
ESC	European Society of Cardiology
CMR	Cardiac Magnetic Resonance
CTA	Computed Tomography Angiography
EAM	Electroanatomical Mapping
