# Supplementary figures and images for: The Marshall Complex in the Human Heart: Embryology, Microanatomy, Autonomic Features and Clinical Implications for Atrial Fibrillation—A State-of-the-Art Narrative Review
Source: J Clin Med. 2025 Nov 27;14(23):8422. doi: 10.3390/jcm14238422 (PMC12693601; doi:10.3390/jcm14238422)

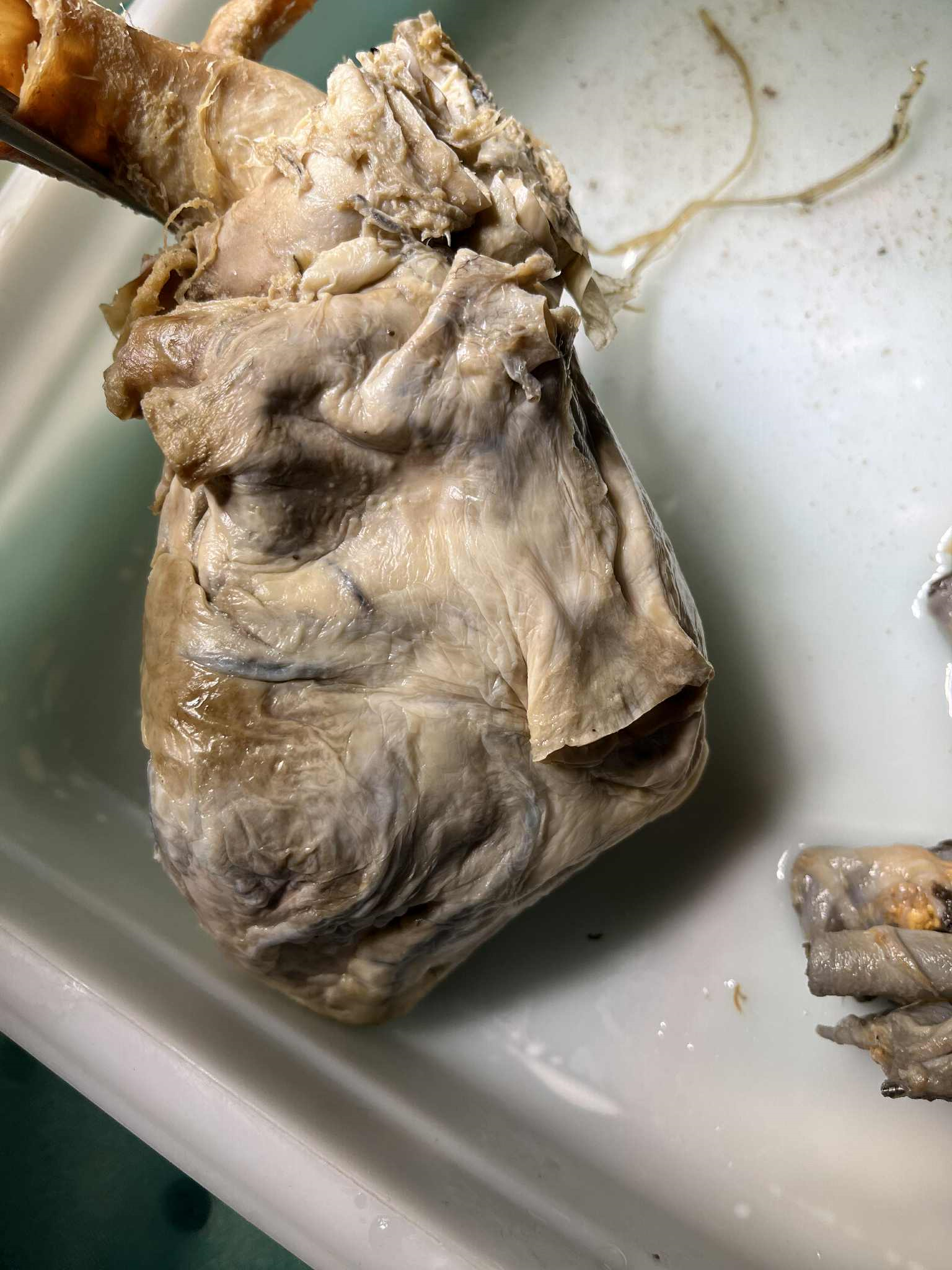

Supplement: Supplementary file 1 [file jcm-14-08422-s001.zip › Figure S1.png]
